# Stevia (*Stevia rebaudiana*) extract ameliorates insulin resistance by regulating mitochondrial function and oxidative stress in the skeletal muscle of *db/db* mice

**DOI:** 10.1186/s12906-023-04033-5

**Published:** 2023-07-24

**Authors:** Jin-Young Han, Miey Park, Hae-Jeung Lee

**Affiliations:** 1 Institute for Aging and Clinical Nutrition Research, Gachon University, Seongnam-Si, 13120 Gyeonggi-Do Korea; 2 Department of Food and Nutrition, College of BioNano Technology, Gachon University, Seongnam-Si, 13120 Gyeonggi-Do Korea

**Keywords:** T2DM, *Stevia rebaudiana*, Muscle fiber size, Insulin resistance, Mitochondrial function, Oxidative stress

## Abstract

**Background:**

Type 2 diabetes mellitus (T2DM), a growing health problem worldwide, is a metabolic disorder characterized by hyperglycemia due to insulin resistance and defective insulin secretion by pancreatic β-cells. The skeletal muscle is a central organ that consumes most of the insulin-stimulated glucose in the body, and insulin resistance can damage muscles in T2DM. Based on a strong correlation between diabetes and muscles, we investigated the effects of stevia extract (SE) and stevioside (SV) on the skeletal muscle of diabetic *db/db* mice.

**Methods:**

The mice were administered saline, metformin  (200 mg/kg/day), SE (200 and 500 mg/kg/day), and SV (40 mg/kg/day) for 35 days. During administration, we checked the levels of fasting blood glucose twice a week and conducted the oral glucose tolerance test (OGTT) and insulin tolerance test (ITT). After administration, we analyzed serum biochemical parameters, triglyceride (TG), total cholesterol (TC), insulin and antioxidant enzymes, and the cross-sectional area of skeletal muscle fibers of *db/db* mice. Western blots were conducted using the skeletal muscle of mice to examine the effect of SE and SV on protein expression of insulin signaling, mitochondrial function, and oxidative stress.

**Results:**

SE and SV administration lowered the levels of fasting blood glucose, OGTT, and ITT in *db/db* mice. The administration also decreased serum levels of TG, TC, and insulin while increasing those of superoxide dismutase (SOD) and glutathione peroxidase (GPx). Interestingly, muscle fiber size was significantly increased in *db/db* mice treated with SE500 and SV. In the skeletal muscle of *db/db* mice, SE and SV administration activated insulin signaling by increasing the protein expression of insulin receptor substrate, Akt, and glucose transporter type 4. Furthermore, SE500 administration markedly increased the protein expression of AMP-activated protein kinase-α, sirtuin-1, and peroxisome proliferator-activated receptor-γ coactivator-1α. SV administration significantly reduced oxidative stress by down-regulating the protein expression of 4-hydroxynonenal, heme oxygenase-1, SOD, and GPx. In addition, SE500 and SV administration suppressed the expression of apoptosis-related proteins in the skeletal muscle of *db/db* mice.

**Conclusion:**

SE and SV administration attenuated hyperglycemia in diabetic mice. Moreover, the administration ameliorated insulin resistance by regulating mitochondrial function and oxidative stress, increasing muscle fiber size. Overall, this study suggests that SE and SV administration may serve as a potential strategy for the treatment of diabetic muscles.

**Supplementary Information:**

The online version contains supplementary material available at 10.1186/s12906-023-04033-5.

## Introduction

Type 2 diabetes mellitus (T2DM) is one of the most common metabolic diseases worldwide [1, 2]. The pathophysiology of T2DM includes disorders of carbohydrate, lipid, and protein metabolism resulting from defects in insulin secretion and action [2, 3]. Insulin resistance leads to the disruption of glucose homeostasis, which increased glucose production in the liver and reduced glucose uptake in skeletal muscle and adipose tissue [2, 4]. Fasting and postprandial hyperglycemia cause secondary complications in various organs, such as the heart, brain, kidneys, and eyes [3–5]. Target drugs for diabetes have been developed, but their side effects and disadvantages are still controversial [6]. Therefore, it is essential to research using natural functional materials with low side effects for diabetes.

Skeletal muscle, a significant determinant of resting metabolic rate, accounts for 40%–50% of the total body weight [7] and consumes approximately 80% glucose stimulated by insulin [8–10]. An essential function of the muscle is to increase glucose transport activity via the insulin signaling cascade [11]. The binding of insulin to the insulin receptor induces insulin receptor substrate (IRS)-1 tyrosine phosphorylation. This results in the activation of Akt phosphorylation, which facilitates the translocation of glucose transporter type 4 (GLUT4) and subsequent glucose uptake into muscle cells [12, 13].

Insulin resistance decreases protein synthesis and increases protein degradation, leading to muscle atrophy and sarcopenia [14, 15]. In skeletal muscle, impaired mitochondrial function causes lipid oxidation, resulting in insulin resistance [16]. AMP-activated protein kinase-α (AMPKα), a central regulator of mitochondrial biogenesis, controls intracellular energy balance and is an essential factor in skeletal muscle atrophy [17, 18]. Deacetylation of peroxisome proliferator-activated receptor-γ coactivator-1α (PGC-1α) plays an essential role in mitochondrial biogenesis, which is regulated by sirtuin-1 (SIRT1) [19]. In addition, hyperglycemia-induced oxidative stress contributes to the reduction in insulin action [20]. The abnormal cell signaling resulting from a decreased ability to eliminate oxidative stress can lead to insulin resistance [21]. Furthermore, a persistent state of oxidative stress induces apoptosis [22], which in itself can also cause muscle atrophy [23, 24].

Stevia (*Stevia rebaudiana*) is a natural sweetener used as a substitute for sucrose worldwide [25]. Stevia leaves have been used as tea and medicines in many parts of the world for centuries [25–28]. Several steviol glycosides, which are four-ring diterpenes, such as stevioside (SV), rebaudioside A, D, C, D, and E, dulcoside A and B, have been identified from stevia leaves [29]. This non-caloric sweetener passes through the body without being metabolized, which is why it is favored bydiet-conscious consumers and can be effectively used to treat diabetes, obesity, and cardiovascular disease [25]. SV, an abundant glycoside found in stevia leaves, is 300 times sweeter than sugar [25, 30]. Several studies have reported the therapeutic benefits of stevia and SV, lowering blood sugar, cholesterol, and triglyceride levels and exhibiting [31–34], antioxidant, anti-inflammatory, and anti-cancerous properties [35–37]. In addition, in vivo studies have investigated the antidiabetic effect of SV, suggesting alleviation of hyperglycemia [33, 38]. However, few studies have focused on whether stevia and SV improve insulin resistance in skeletal muscle, essential for insulin-stimulated glucose absorption. Therefore, in this study, we investigated the effect of stevia extract (SE) and its glycoside, SV, on insulin resistance and improvement mechanism in skeletal muscle in diabetic *db/db* mice.

In this study, we used *db/db* mice with severe obesity caused by leptin receptor defects to model T2DM [39]. Metformin, a T2DM medication, was used as a positive control (PC) verifying a significant reduction in blood glucose in mice [40]. This study shows that the effect of SE and SV administration on insulin resistance in skeletal muscle of diabetic mice will occur through the regulation of mitochondrial function and oxidative stress, and suggests that SE and SV administration may improve diabetic muscles compared to metformin administration.

## Materials and methods

### Materials

*Stevia rebaudiana* was registered in plant variety protection right as grant No. 6485 by Korea Seed & Variety Service in 2017, according to Plant Variety Protection Act (application No. 2015–257). Experimental research on plants was performed following the relevant guidelines and legislation [41]. Lyophilized powder of stevia leaves was obtained from Pharminogen Co. (Yongin, Kyunggi-do, Korea). Briefly, stevia leaves were dried and extracted with water at 100 °C for 3 h using a heating mantle. Next, the extract was filtered through 500 mesh filter and concentrated using vacuum evaporation. The concentrate was lyophilized to remove moisture entirely and stored at -20 °C in a powder. Stevioside (C_38_H_60_O_18_) was purchased from ChemFaces Biochemical Co. Ltd. (Wuhan, China).

### Animal studies

Eight-week-old BKS.Cg-Dock7^m^ + / + Leprdb/J (Heterozygous for Dock7^m^/ Heterozygous for *Leprdb*) (*db/m* + , negative controls) and *db/db* mice (BKS.Cg-Dock7^m^ + / + *Leprdb*/J, homozygote) were obtained from Jackson Laboratories (Sacramento, CA, USA). Mice were allowed to adjust to a controlled environment with a 12 h light/dark cycle at 20–25 °C for two weeks. Following the acclimatization period, six non-diabetic mice (*db/m* +) were assigned to the control group, and 30 diabetic mice (*db/db*) were randomly divided into six groups as follows: (1) Negative control (N +) group, non-diabetic mice + saline; (2) Normal control (NC) group, diabetic mice + saline; (3) PC group, diabetic mice + metformin (200 mg/kg BW, p.o.); (4) SE200 group, diabetic mice + SE (SE, 200 mg/kg BW, p.o.); (5) SE500 group, diabetic mice + SE (500 mg/kg BW, p.o.); (6) SV group, diabetic mice + stevioside (SV, 40 mg/kg BW, p.o.). Saline, metformin, SE, and SV were administered by oral gavage daily for 35 days (Figure S1). Mice were weighed twice a week. All animal experiments were performed following the Guide for the Care and Use of Laboratory Animals and were approved by Gachon University (GI-ACUC-R2020012).

### Fasting blood glucose levels (FBGLs), oral glucose tolerance test (OGTT), and insulin tolerance test (ITT)

Mice fasted for 3 h and the FBGLs were measured using Accu-Chek Performa (Roche Diagnostics Korea Co., Ltd., Seoul, Korea). Blood was collected from the tail vein of each mouse. On day 21 of administration, overnight-fasted mice were orally administered glucose (Dai Han Pharm. Co. Ltd., Seoul, Korea) at a concentration of 2 g/kg BW. Blood glucose levels were measured at 0, 15, 30, 60, and 120 min after oral glucose gavage. In addition, ITT was performed on day 35 in mice following fasting for 3 h. Mice were administered an intraperitoneal injection of Humulin (1 U/kg BW; Eli Lilly, Indianapolis, IN, USA) and the blood glucose level was checked at 0, 15, 30, and 60 min. Blood glucose and insulin tolerance levels were determined using the area under the curve (AUC).

### Biochemical analysis

Serum triglyceride (TG) and total cholesterol (TC) levels were determined using commercial kits (Asan Pharm, Seoul, Korea), and serum insulin level was measured using an enzyme-linked immunosorbent assay (ELISA) kit (Thermo Fisher, Waltham, MA, USA). The homeostatic model assessment for insulin resistance (HOMA-IR) was calculated using the following formula: $\mathrm{HOMA}-\mathrm{IR}=\{\mathrm{fasting insulin}\left[\frac{\mathrm{U}}{\mathrm{mL}}\right]\times \mathrm{fasting glucose}\left[\frac{\mathrm{mg}}{\mathrm{dL}}\right]\}/405$ [42]. The concentration of superoxide dismutase (SOD) and glutathione peroxidase (GPx) in mouse serum was measured using Bluegene ELISA kits (Shanghai Bluegene Biotech Co. Ltd., Shanghai, China). The biochemical experiments were conducted according to the manufacturer’s recommendations.

To determine malondialdehyde (MDA) levels, liver homogenates dissolved in 1.15% KCl (Sigma-Aldrich, St Louis, MO) were obtained from one portion of 0.05 g liver of each mouse. Then, the samples were blended with a mixed solution containing 8.1% sodium dodecyl sulfate (SDS) (iNtRON Biotechnology, Seongnam, Korea) 200 μL, 20% acetic acid (Daejung Chemical, Suwon, Korea), 1.5 mL 0.8% thiobarbituric acid (TBA) (Sigma-Aldrich, St Louis, MO) 1.5 mL, distilled water 700 μL. A 5 ml volume of n-butanol (SAMCHUN, Gyeonggi-do, Korea) and 1 ml volume of distilled water were added to mixtures, followed by incubating in a 95 °C water bath for 30 min and ice for 10 min. After centrifuging at 4,000 rpm for 10 min, MDA levels of each supernatant were measured at 532 nm.

### Hematoxylin and eosin (H&E) staining and analysis of the cross-sectional area of skeletal muscle fibers

Skeletal muscle tissue samples of 3 mice in each group were fixed in 10% formalin (Sigma-Aldrich, St. Louis, MO, USA). The samples were embedded in paraffin and stained with H&E. Stained sections were visualized using an Olympus Provis AX70 microscope (Olympus, Tokyo, Japan). To analyze muscle fiber size, the cross-sectional area of the skeletal muscle in mice was calculated using ImageJ software (rsb.info.nih.gov/ij).

### Western blot analysis

Gastrocnemius tissue (20 mg) was homogenized in PRO-PREP™ solution (iNtRON Biotechnology, Seongnam, Korea) containing a phosphatase inhibitor (Thermo Fisher) and incubated for 30 min on ice. Following lysis, the tissue suspension was centrifuged at 13,000 × *g* for 5 min at 4 °C to obtain the supernatant. Nuclear and cytoplasmic fractions were isolated using NE-PER Nuclear and Cytoplasmic Extraction Reagents (Thermo Fisher) according to the manufacturer’s instructions. Protein concentrations were measured at 595 nm using the PRO-MEASURE™ solution (iNtRON Biotechnology). Concentrations were calculated according to the manufacturer’s instructions.

Equal amounts of total protein (30 μg) were electrophoresed by sodium dodecyl sulfate polyacrylamide gel electrophoresis (SDS-PAGE) and subsequently transferred onto a polyvinylidene difluoride (PVDF) membrane. The membranes were blocked with 5% skim milk for 1 h and incubated with the following primary antibodies: p-Akt, Akt, p-IRS, IRS, p-AMPKα, AMPKα, citrate synthase (CS) (Cell Signaling Technology, Beverly, MA, USA, 1:1000), SIRT1, 4-hydroxynonenal (4-HNE), Bax, Bcl**-**2 (Abcam, Cambridge, MA, USA, 1:1000), Lamin B1 (Abcam, 1:10,000), α-tubulin (Abcam, 1:5000), heme oxygenase 1 (HO-1), SOD, GPx (Santa Cruz Biotechnology, Santa Cruz, CA, USA, 1:200), PGC-1α (Bioss Antibodies, Woburn, MA, USA, 1:1000), and GLUT4 (Thermo Fisher, 1:1000). After incubation with secondary antibodies for 1 h, specific bands were visualized using an enhanced chemiluminescence method (iNtRON Biotechnology) and a Quant LAS 500 system (GE Healthcare Bio-Sciences AB, Björkgatan, Uppsala, Sweden).

### Statistical analysis

GraphPad Prism 5.03 (GraphPad Software Inc., La Jolla, CA, USA) was used for statistical analysis using one-way analysis of variance (ANOVA), followed by Tukey’s *post-hoc* test. Data were presented as the mean ± standard deviation (SD). Statistical significance was set at *p* < 0.05.

## Results

### Effect of SE on the body, subcutaneous fat, and gastrocnemius weights of *db/db* mice

**Fig. 1:**
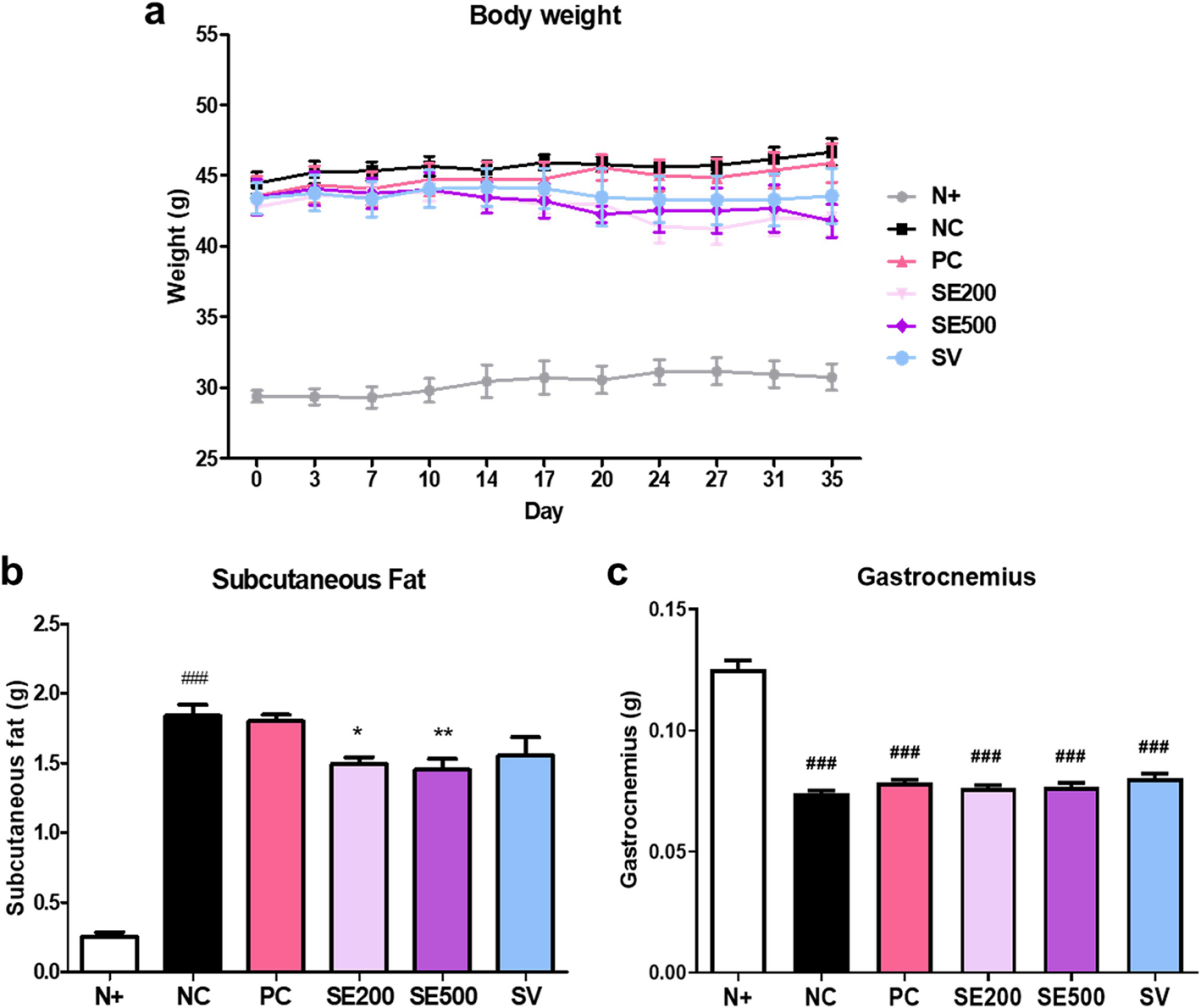
Effects of SE on body, subcutaneous fat, and gastrocnemius weights of *db/db* mice. **a** Body weight, (**b**) subcutaneous fat weight, and (**c**) gastrocnemius weight. The data were presented as mean ± SD. N + : *db/m* +  + saline; NC: *db/db* + saline; PC: *db/db* + metformin; SE200: *db/db* + stevia extract 200 mg/kg BW; SE500: *db/db* + stevia extract 500 mg/kg BW; SV: *db/db* + stevioside 40 mg/kg BW. ^###^ *p* < 0.001 vs. N + ; ^**^ *p* < 0.01, ^*^ *p* < 0.05 vs. NC

All mice were weighed twice a week during the administration of the compounds. On the last day of the experiment, differences were noted between the diabetic groups, except for the N + mice. However, no significant difference was observed (Fig. 1a). N + mice had markedly lower subcutaneous fat than diabetic NC mice. Subcutaneous fat weight was lower in SE-treated mice than in NC mice. However, the SV group did not show a significant difference (Fig. 1b). The gastrocnemius weight was the highest in N + mice, and there was no difference between the diabetic groups (Fig. 1c). In summary, subcutaneous fat weight decreased, but gastrocnemius weight remained unchanged in SE-treated mice. Thus, in subsequent experiments, we focused on the effect of SE on skeletal muscle in diabetic mice.

### Effect of SE on FBGLs, OGTT, and ITT in *db/db* mice

**Fig. 2:**
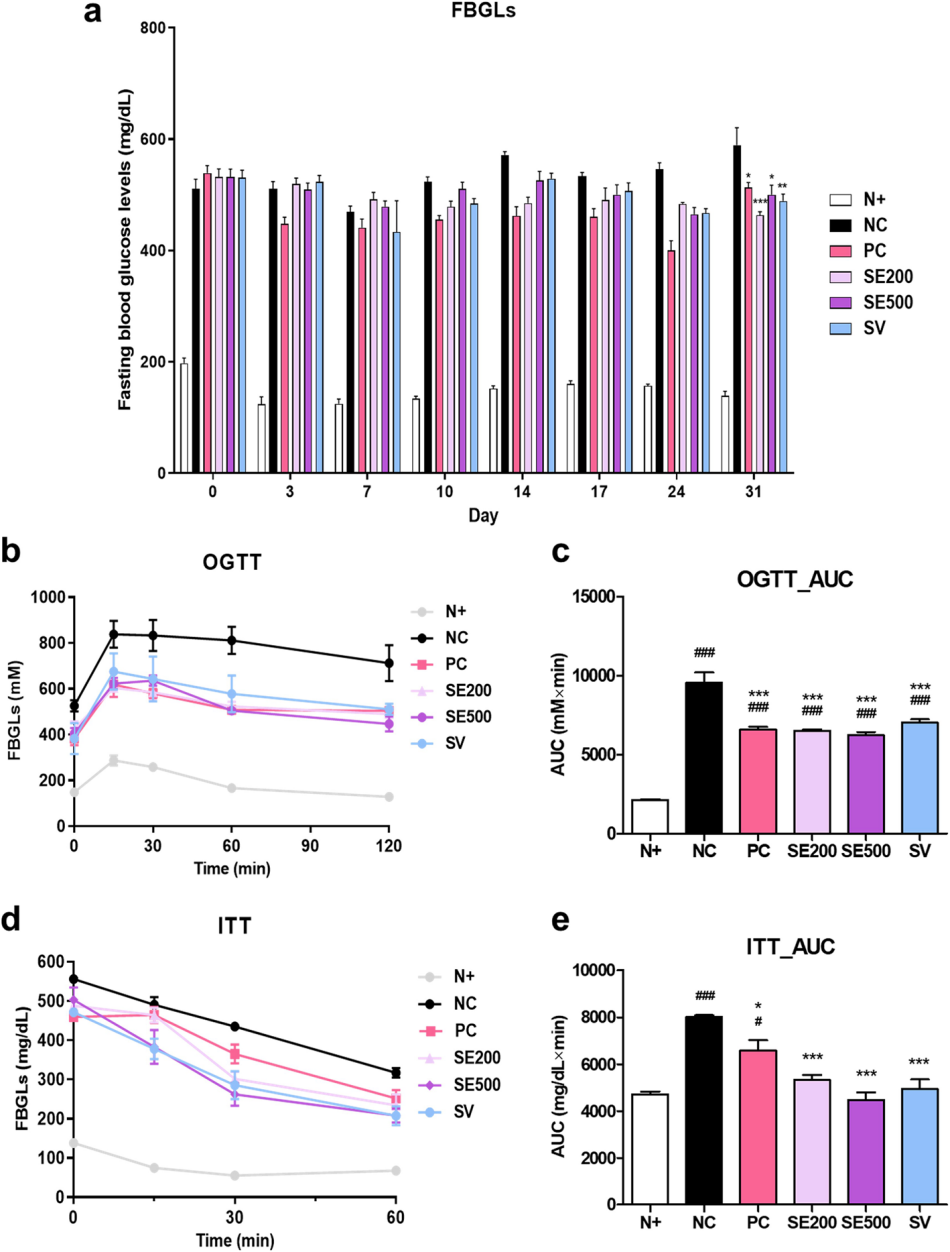
Effects of SE on fasting blood glucose levels (FBGLs), oral glucose tolerance test (OGTT), and insulin tolerance test (ITT) in *db/db* mice. **a** FBGLs, (**b**) OGTT levels, (**c**) area under the curve (AUC) of OGTT, (**d**) ITT levels, and (**e**) AUC of ITT. The data were presented as mean ± SD. N + : *db/m* +  + saline; NC: *db/db* + saline; PC: *db/db* + metformin; SE200: *db/db* + stevia extract 200 mg/kg BW; SE500: *db/db* + stevia extract 500 mg/kg BW; SV: *db/db* + stevioside 40 mg/kg BW. ^###^ *p* < 0.001, ^#^ *p* < 0.05 vs. N + ; ^***^ *p* < 0.001, ^**^ *p* < 0.01, ^*^ *p* < 0.05 vs. NC

We measured the FBGLs on days 0, 3, 7, 10, 14, 17, 24, and 31. At the beginning of the experiment, there were no significant differences in the blood glucose levels of mice in the experimental groups, except the N + mice. However, on day 31, FBGLs in the experimental mice declined significantly (Fig. 2a). To investigate glucose tolerance, we performed OGTT and ITT on days 21 and 35, respectively. Compared to *db/db* mice, mice in the experimental groups showed improved glucose tolerance, which was also noted in the AUC of the OGTT (Fig. 2b and c). A similar pattern was observed in the ITT, where the experimental mice showed improved glucose disposal compared to the NC mice (Fig. 2d and e). The higher dose of SE appeared to be most effective for glucose disposal and insulin action.

### Effect of SE on biochemical markers associated with diabetes in *db/db* mice

**Fig. 3:**
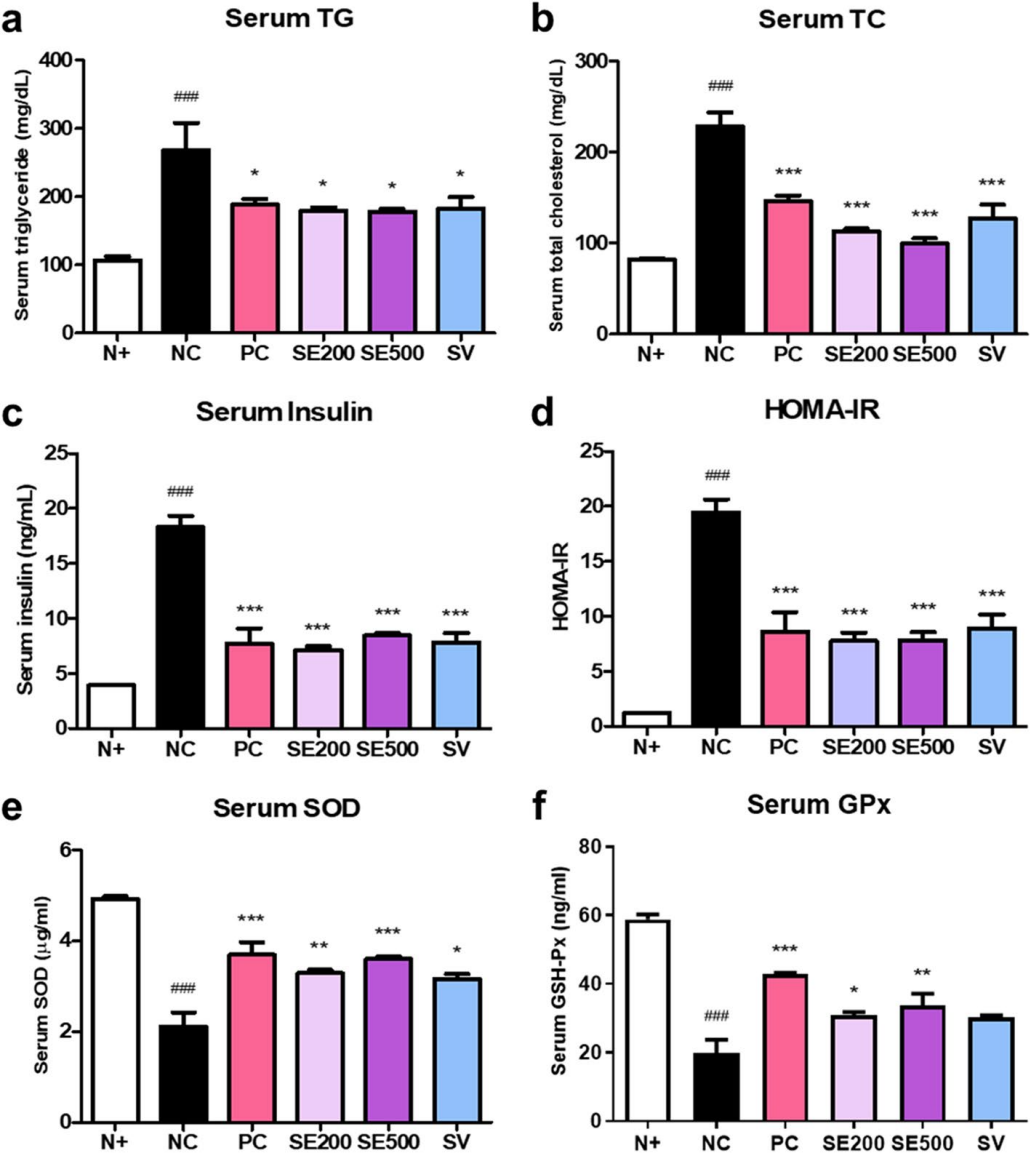
Effects of SE on biochemical markers associated with diabetes in *db/db* mice. Serum (**a**) TG and (**b**) TC levels. **c** Serum insulin and (**d**) HOMA-IR levels. Serum (**e**) SOD and (**f**) GPx levels. The data were presented as mean ± SD. N + : *db/m* +  + saline; NC: *db/db* + saline; PC: *db/db* + metformin; SE200: *db/db* + stevia extract 200 mg/kg BW; SE500: *db/db* + stevia extract 500 mg/kg BW; SV: *db/db* + stevioside 40 mg/kg BW. ^###^ *p* < 0.001 vs. N + ; ^***^ *p* < 0.001, ^**^ *p* < 0.01, ^*^ *p* < 0.05 vs. NC

To examine the biochemical markers related to diabetes, we measured the serum TG and TC levels using the blood from the tail vein of mice. NC mice showed higher serum TG and TC levels than N + mice. TG levels were reduced in the experimental mice (Fig. 3a). Consistent with the TG results, serum TC levels were also reduced in the experimental mice, and the SE500 mice showed the lowest TC levels (Fig. 3b). Next, serum insulin and HOMA-IR levels to determine whether SE administration affects serum insulin levels. NC mice displayed elevated serum insulin concentrations compared to N + mice. The experimental mice exhibited significantly reduced serum insulin and HOMA-IR levels (Fig. 3c and d). Antioxidant enzyme content is closely related to diabetes, which is a condition attributable to excessive oxidative stress. To assess the effect of stevia on antioxidant enzyme levels, we measured the levels of SOD and GPx, which are representative antioxidant enzymes, in mice serum. NC mice exhibited lower serum SOD and GPx levels than N + mice (Fig. 3e and f). In contrast, the experimental mice showed significantly higher antioxidant enzyme levels than the NC mice, except for GPx in the SV mice. In addition, to investigate oxidative stress caused by lipid peroxides, we measured hepatic MDA levels in diabetic mice. PC mice showed significant reductions in hepatic MDA levels. In mice groups with stevia treatments, SE500 and SV mice had significantly lower MDA levels compared to NC mice (Figure S2).

### Effect of SE on the size of skeletal muscle fibers in *db/db* mice

**Fig. 4:**
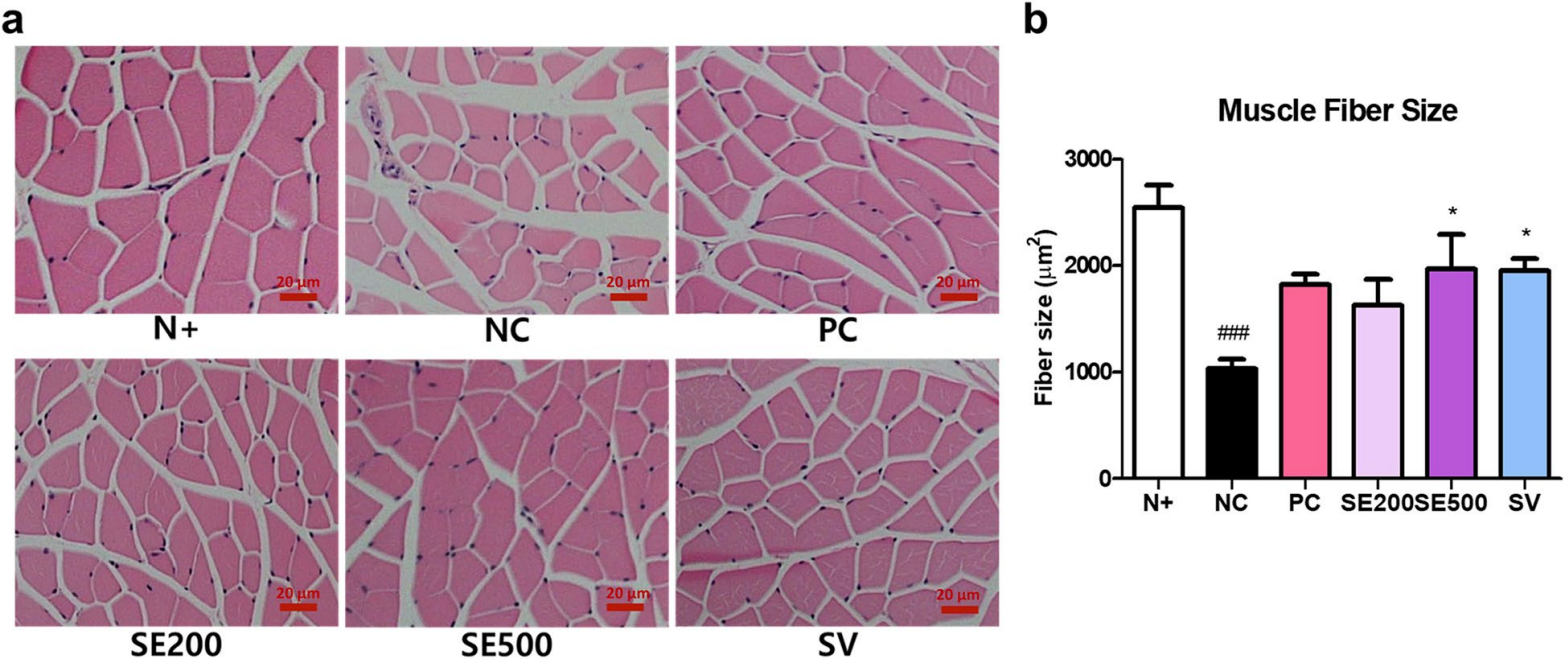
Effects of SE on skeletal muscle fiber size in *db/db* mice. **a** Cross-sectional area of muscle fibers, and (**b**) muscle fiber size. The scale bar is 20 μm. The data were presented as mean ± SD. N + : *db/m* +  + saline; NC: *db/db* + saline; PC: *db/db* + metformin; SE200: *db/db* + stevia extract 200 mg/kg BW; SE500: *db/db* + stevia extract 500 mg/kg BW; SV: *db/db* + stevioside 40 mg/kg BW. ^###^ *p* < 0.001 vs. N + ; ^*^ *p* < 0.05 vs. NC

As mentioned before, SE administration decreased subcutaneous fat weight but did not affect gastrocnemius weight in mice. Therefore, we examined the morphological changes in skeletal muscle by H&E staining of cross-sections of skeletal muscle tissues. Muscle fiber size was significantly decreased, and degenerated fibers were observed in NC mice compared to those in N + mice (Fig. 4a and b). The cross-sectional areas of SE500 and SV mice exhibited a significant increase compared to that of NC mice.

### Effect of SE on insulin signaling in skeletal muscle of *db/db* mice

**Fig. 5:**
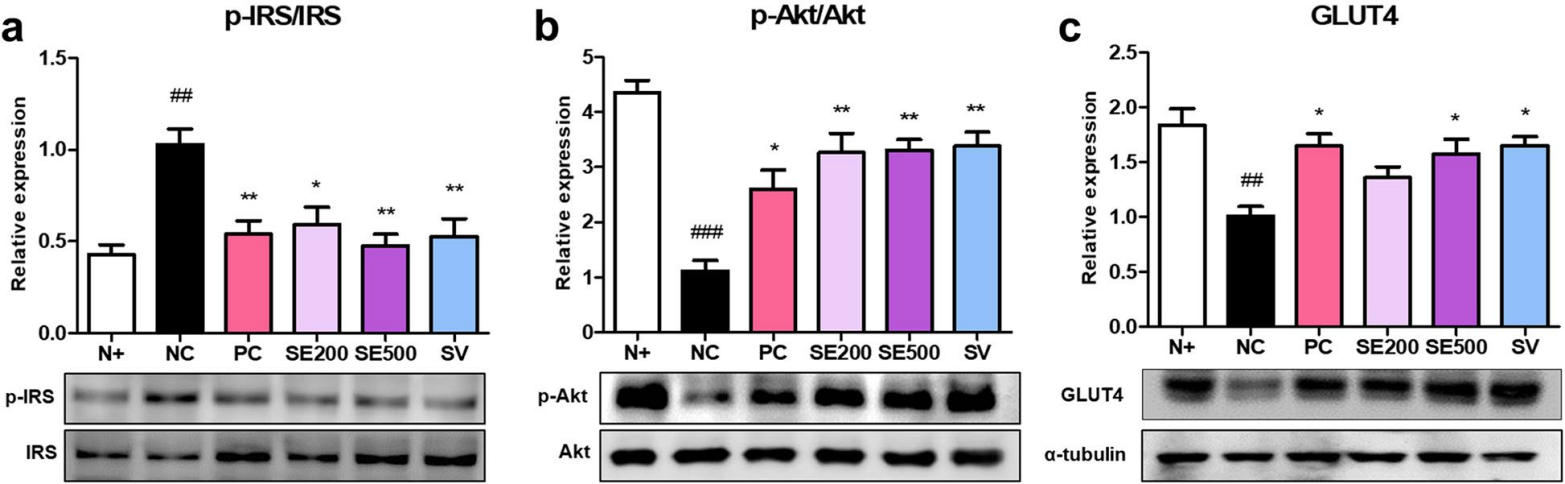
Effects of SE on the expression of insulin signaling proteins in skeletal muscle of *db/db* mice. The protein expression of (**a**) p-IRS/IRS ratio, (**b**) p-Akt/Akt ratio, and (**c**) GLUT4. All results were expressed following normalization with α-tubulin. The data were presented as mean ± SD. N + : *db/m* +  + saline; NC: *db/db* + saline; PC: *db/db* + metformin; SE200: *db/db* + stevia extract 200 mg/kg BW; SE500: *db/db* + stevia extract 500 mg/kg BW; SV: *db/db* + stevioside 40 mg/kg BW. ^###^ *p* < 0.001, ^##^* p* < 0.01 vs. N + ; ^**^ *p* < 0.01, ^*^ *p* < 0.05 vs. NC

To determine whether SE administration affects insulin metabolism in diabetic skeletal muscles, we analyzed the levels of proteins related to the insulin signaling pathway using western blotting. Serine phosphorylation of IRS, which indicates insulin resistance, was the highest in NC mice. The experimental groups showed decreased p-IRS/IRS ratios (Fig. 5a). The p-Akt/Akt ratio was lower in the NC mice than in the N + mice. In contrast, the p-Akt/Akt ratio was higher in the experimental mice than in the NC mice (Fig. 5b). Similarly, GLUT4 expression was the lowest in NC mice. There was no significant difference in GLUT4 levels between SE200 mice, but it was higher compared to that in NC mice (Fig. 5c). GLUT4 expression was significantly higher in PC, SE500, and SV-treated mice. These data suggest that the administration increases glucose transport into insulin target tissues.

### Effect of SE on mitochondrial activity in skeletal muscle of *db/db* mice

**Fig. 6:**
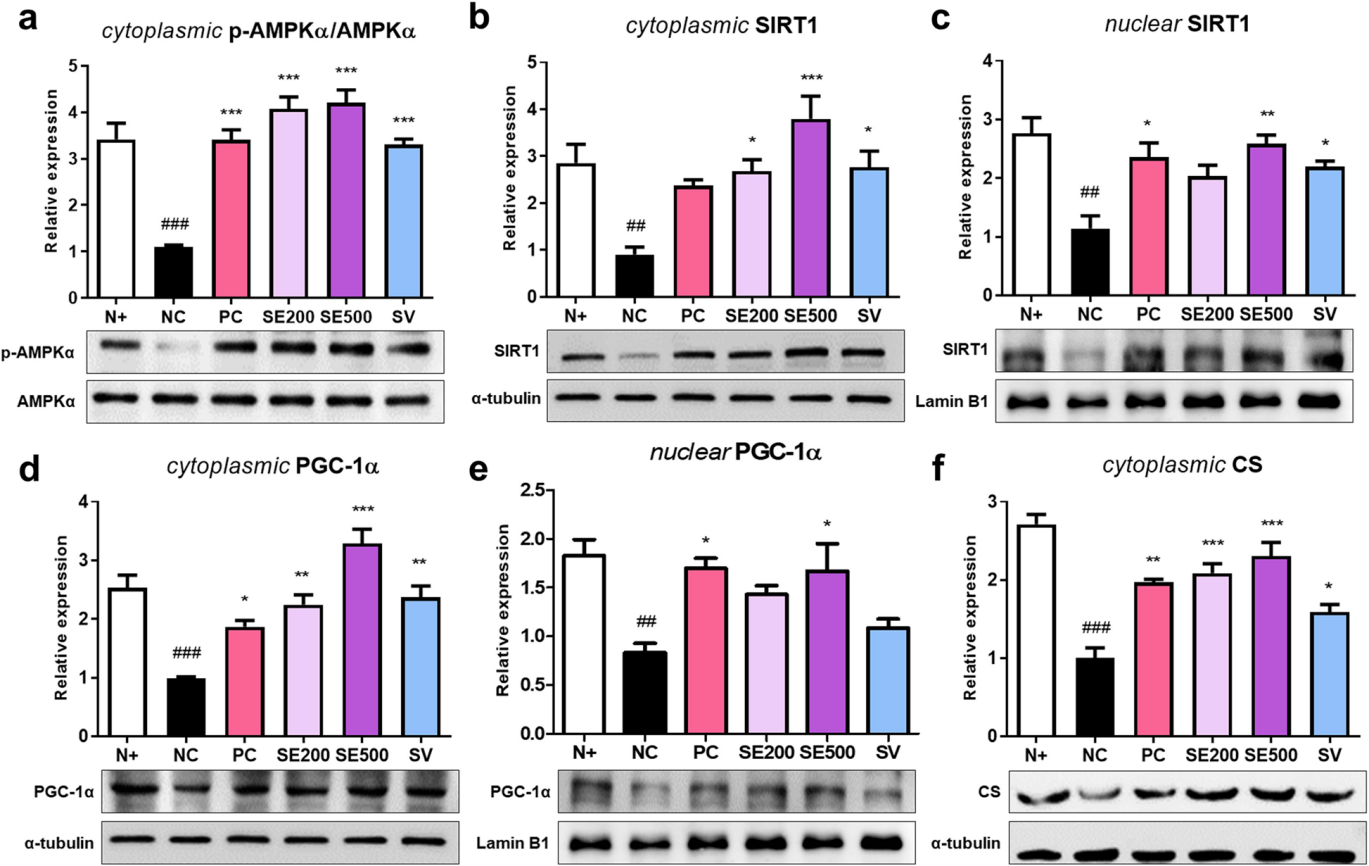
Effects of SE on the expression of mitochondrial activity proteins in skeletal muscle of *db/db* mice. The protein expression of (**a**) cytoplasmic p-AMPKα/AMPKα ratio, (**b**) cytoplasmic SIRT1, (**c**) nuclear SIRT1, (**d**) cytoplasmic PGC-1α, (**e**) nuclear PGC-1α, and (**f**) cytoplasmic CS. All results were expressed following normalization with α-tubulin or Lamin B1. The data were presented as mean ± SD. N + : *db/m* +  + saline; NC: *db/db* + saline; PC: *db/db* + metformin; SE200: *db/db* + stevia extract 200 mg/kg BW; SE500: *db/db* + stevia extract 500 mg/kg BW; SV: *db/db* + stevioside 40 mg/kg BW. ^###^ *p* < 0.001, ^##^ *p* < 0.01 vs. N + ; ^***^ *p* < 0.001, ^**^ *p* < 0.01, ^*^ *p* < 0.05 vs. NC

We investigated the effect of SE administration on energy metabolism in diabetic skeletal muscle by measuring the level of energy metabolism-associated proteins in the cytoplasm and nucleus. As shown in Fig. 6a, NC mice exhibited a significantly reduced cytoplasmic p-AMPKα/AMPKα ratio compared to N + mice. AMPKα phosphorylation was markedly increased in the experimental groups compared to NC mice. The expression of cytoplasmic and nuclear SIRT1, which is enhanced by AMPKα activation, was the lowest in NC mice, whereas it was elevated in SE and SV mice (Fig. 6b and c). PGC-1α, a key mediator of mitochondrial biogenesis, also showed a similar expression to AMPKα and SIRT1 in the cytoplasm, considerably increasing SE500 mice (Fig. 6d). The expression of nuclear PGC-1α was significantly increased only in PC and SE500 mice (Fig. 6e). To determine whether SE administration affects mitochondrial content, we measured the expression of cytoplasmic CS. PC, SE, and SV-treated mice displayed increased CS expression compared to NC mice (Fig. 6f). These results suggest that mitochondrial activity is enhanced in skeletal muscle of diabetic mice treated with a high dose of SE.

### Effect of SE on oxidative stress and apoptosis-related proteins in skeletal muscle of *db/db* mice

**Fig. 7:**
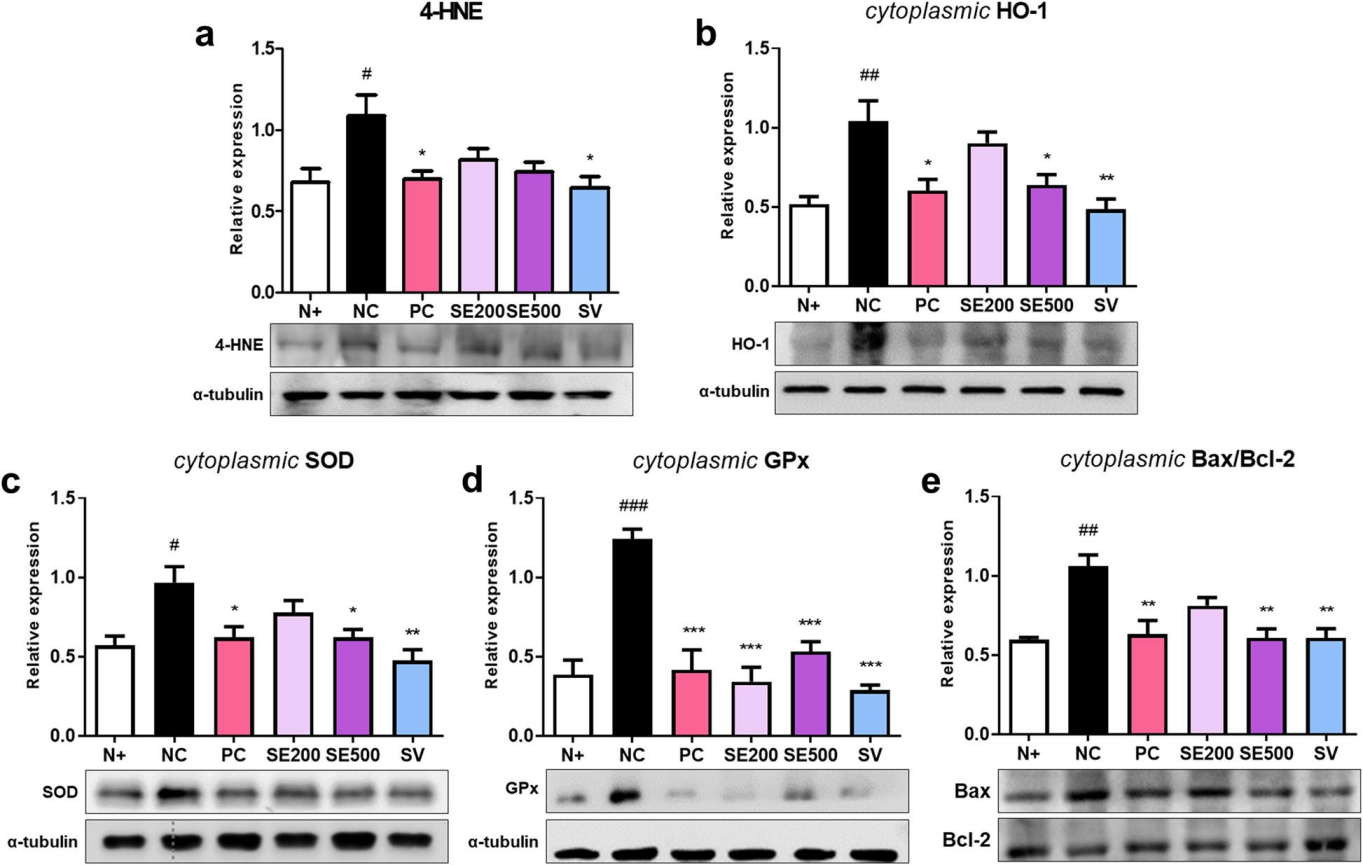
Effects of SE on the expression of proteins related to oxidative stress and apoptosis in skeletal muscle of *db/db* mice. The protein expression of (**a**) 4-HNE, (**b**) cytoplasmic HO-1, (**c**) cytoplasmic SOD, (**d**) cytoplasmic GPx, and (**e**) cytoplasmic Bax/Bcl-2 ratio. All results were expressed following normalization with α-tubulin. The data were presented as mean ± SD. N + : *db/m* +  + saline; NC: *db/db* + saline; PC: *db/db* + metformin; SE200: *db/db* + stevia extract 200 mg/kg BW; SE500: *db/db* + stevia extract 500 mg/kg BW; SV: *db/db* + stevioside 40 mg/kg BW. ^###^ *p* < 0.001, ^##^ *p* < 0.01, ^#^ *p* < 0.05 vs. N + ; ^***^ *p* < 0.001, ^**^ *p* < 0.01, ^*^ *p* < 0.05 vs. NC

We analyzed the expression of proteins related to oxidative stress in diabetic skeletal muscles. The NC mice showed the highest level of 4-HNE, a typical lipid peroxidation aldehyde that can cause mitochondrial damage and apoptosis [43]. SE-treated mice did not show a significant decrease in 4-HNE expression compared to NC mice, whereas the PC and SV mice exhibited significantly reduced 4-HNE levels (Fig. 7a). Next, we analyzed the expression of cytoplasmic proteins that protect against oxidative stress. HO-1, which alleviates oxidative damage, was significantly higher in NC mice than in N + mice and was decreased in PC, SE500, and SV mice (Fig. 7b). In addition, NC mice exhibited high expression of SOD and GPx, which are critical antioxidant proteins that scavenge ROS [44]. The expression of SOD showed significant differences only in PC, SE500, and SV mice compared to that in NC mice (Fig. 7c), and GPx was decreased in all experimental mice (Fig. 7d). Overall, among the treatments, SV treatment appeared to protect against oxidative stress.

Given the chronic oxidative stress conditions, we examined the ratio of the apoptosis-related factors Bax and Bcl**-**2 in the diabetic skeletal muscle. The NC mice displayed an elevated Bax/Bcl**-**2 ratio compared to N + and experimental mice, except for SE200, which exhibited a significantly decreased ratio compared to the NC mice (Fig. 7e).

## Discussion

In the current study, we investigated the effects of SE and SV administration on insulin resistance and its underlying mechanism in the skeletal muscle of diabetic mice. First, treatment of SE and SV significantly reduced hyperglycemia. Additionally, the treatment raised the levels of antioxidant enzymes while decreasing serum TG, TC, and insulin levels. The administration of metformin showed the same results. Interestingly, only mice given SE500 and SV experienced an increase in muscle fiber size. In the skeletal muscle of diabetic mice, PC, SE500, and SV administration triggered insulin signaling. In particular, SE500 administration had a notable impact on elevated mitochondrial activity. Furthermore, the study demonstrated that PC and SV treatments were associated with lowering oxidative stress. Overall, this study found that supplying SE and SV to db/db mice’s skeletal muscles ameliorated their insulin resistance and was associated with regulating mitochondrial function and oxidative stress.

The incidence of T2DM, a severe metabolic disorder, is increasing worldwide. T2DM, characterized by high blood glucose and insulin dysfunction, can damage the cardiovascular system, spleen, retina, and skeletal muscles [2, 5, 45]. Given the side effects of anti-diabetic drugs, managing diabetes with herbal medicines based on plants and natural phytochemicals such as alkaloids, polysaccharides, and flavonoids is gaining more attention [46]. In the present study, we investigated the effect of stevia, a natural sweetener, on diabetes in *db/db* mice.

Stevia has been used as a sweetener for decades and has been reported to have various health benefits. Some studies have investigated the health risks of stevia, including the allergenic potential of purified stevia in rats [47] and its effect in reducing fertility in female rats [48]. Nevertheless, stevia and SV which have non-caloric properties are consistently proposed as a sugar substitute in obesity and diabetes [30, 31, 33, 49, 50]. In the present study, PC, SE, and SV administration significantly suppressed the increased blood glucose, serum TG, and serum TC levels in *db/db* mice. In addition, we performed an OGTT to monitor peripheral disposal after oral glucose loading and insulin secretion over time [51]. The AUC of the OGTT in the treated mice was significantly lower than that in the NC group, indicating greater glucose intolerance. The results of ITT, reflecting whole-body insulin action, showed a decrease in treated mice, similar to the results of the OGTT. NC mice show hyperinsulinemia, one of the symptoms of diabetes. SE administration has been reported to reduce serum insulin levels and HOMA-IR, an indicator of hepatic insulin action [52].

Our results manifested that SE500 and SV administration increased muscle fiber size while ameliorating muscle damage. It is unclear why PC mice treated with metformin did not exhibit a substantial increase; nonetheless, a recent study found that metformin causes induces atrophy by regulating myostatin [53]. To determine whether the effects of SE and SV on muscle are related to insulin action, we investigated insulin signaling in the skeletal muscle of *db/db* mice. Insulin binds to IR, resulting in glucose uptake and tyrosine kinase activation, leading to IRS phosphorylation. Subsequent activation of the PI3K/Akt pathway results in the translocation of GLUT4 to the membrane, thereby mediating glucose transport [54]. One study substantiated that SV activated IR/IRS-1/Akt/GLUT 4 signaling, promoting glucose uptake in the gastrocnemius muscle of diabetic rats [55]. In this study, consistent with the metformin group, SE and SV administration was found to upregulate the expression of proteins related to insulin activity. Insulin resistance reduces muscle quality and strength, resulting in muscle atrophy [56]. It is necessary to confirm the significant overlap in molecular pathways in which sarcopenia and muscle IR are abnormally regulated [57]. Previous studies have reported that treatment with rosiglitazone and anti-myostatin antibodies increased insulin sensitivity and suppressed muscle protein degradation, and increased skeletal muscle mass and strength in mice [58, 59]. Likewise, our data showed that SE and SV administration improved insulin resistance, increasing muscle fiber size and alleviating muscle damage. Meanwhile, insulin resistance is closely associated with mitochondrial dysfunction [60]. One study reported that the enzymatic activity of mitochondrial complexes involved in oxidative phosphorylation was reduced by approximately 40% in human skeletal muscle in T2DM [61]. In mitochondrial biogenesis, PGC-1α is essential in increasing cellular ATP [62], and SIRT1-mediated PGC-1α functions to promote metabolic adaptations in tissue [63]. In the current study, PC, SE, and SV administration upregulated the protein expression of p-AMPKα/AMPKα ratio and SIRT1 and consequently activated the PGC-1α in the skeletal muscle of *db/db* mice. In particular, a high dose of SE effectively activated mitochondrial function. To further support the effect on mitochondria, we measured the protein expression levels of CS. PC, SE, and SV administration increased CS levels, which increased mitochondrial function and content [64]. Consequently, the administration improved insulin resistance by activating the AMPK/SIRT1/PGC-1α pathway in the skeletal muscle of *db/db* mice.

The activity of antioxidant proteins increases under high oxidative stress [65], such as T2DM, Alzheimer’s disease, and cardiovascular disease. SOD and GPx are key cellular antioxidant enzymes that scavenge toxic free radicals and neutralize ROS [44]. Therefore, we assessed the expression of proteins related to oxidative stress, which can affect impaired insulin action [20]. NC mice exhibited increased HO-1, SOD, and GPx protein levels, indicating that severe diabetic conditions induce oxidative stress. In contrast, PC, SE500, and SV administration suppressed the expression of these proteins. In particular, SV administration showed a strong antioxidant effect, similar to the results of other studies [20, 66, 67]. Furthermore, we investigated aberrant apoptosis, which may be caused by excessive free radicals [68]. The Bax/Bcl**-**2 ratio, a key role in the apoptotic pathway, was increased in diabetic mice and decreased in PC, SE500, and SV mice. These findings indicated that SE500 and SV administration decreased oxidative stress and apoptosis, which enhanced insulin sensitivity.

Several studies have been reported [69–71] on the antihyperglycemic effects of stevia, or SV, in general adults. We used the skeletal muscle of diabetic mice to examine their antihyperglycemic mechanisms. According to a recent study, SV attenuated insulin resistance in rats fed a high-fat diet by activating the insulin signaling pathway in their muscles [55]. We did, however, confirm the effects of SE and SV together and found that they activated insulin signaling while also controlling mitochondrial function and oxidative stress. However, given that this study only involved a small number of animals, translating its findings into clinical practice is challenging. Future research on deeper mechanisms, including inflammation and fatty acid-induced insulin resistance, using sufficiently large numbers of animals is needed in order to completely comprehend the effects of SE and SV in diabetic muscle. Furthermore, future research may also need to be tailored for the muscles of patients with type 2 diabetes.

In this study, SE and SV administration improves insulin resistance by activating mitochondrial function through the activation AMPK/SIRT1/PGC-1α pathway and reducing oxidative stress, which increases muscle fiber size and alleviates degenerated fibers in *db/db* mice. Therefore, our data suggest that SE and SV may be potential nutraceuticals for the management of diabetic muscle.

## Supplementary Information

**Additional file 1.** **FigureS1.** The schedule for animal experiments.

**Additional file 2.** **FigureS2.** Hepatic MDA levels in *db/db* mice.

**Additional file 3.** Original images for western blots.

## Data Availability

All data and results of the current study are available from the corresponding authors upon reasonable request.

## Abbreviations

T2DM: Type 2 diabetes mellitus
IRS: Insulin receptor substrate
GLUT4: Glucose transporter type 4
AMPK α: AMP-activated protein kinase-α
PGC-1α: Proliferator-activated receptor-γ coactivator-1α
SIRT1: Sirtuin-1
SE: Stevia extract
SV: Stevioside
FBGLs: Fasting blood glucose levels
OGTT: Oral glucose tolerance test
ITT: Insulin tolerance test
TG: Triglyceride
TC: Total cholesterol
HOMA-IR: Homeostatic model assessment for insulin resistance
SOD: Superoxide dismutase
GPx: Glutathione peroxidase
CS: Citrate synthase
4-HNE: 4-Hydroxynonenal
HO-1: Heme oxygenase-1
