# Supplementary figures and images for: Stevia (Stevia rebaudiana) extract ameliorates insulin resistance by regulating mitochondrial function and oxidative stress in the skeletal muscle of db/db mice
Source: BMC Complement Med Ther. 2023 Jul 24;23:264. doi: 10.1186/s12906-023-04033-5 (PMC10367355; doi:10.1186/s12906-023-04033-5)

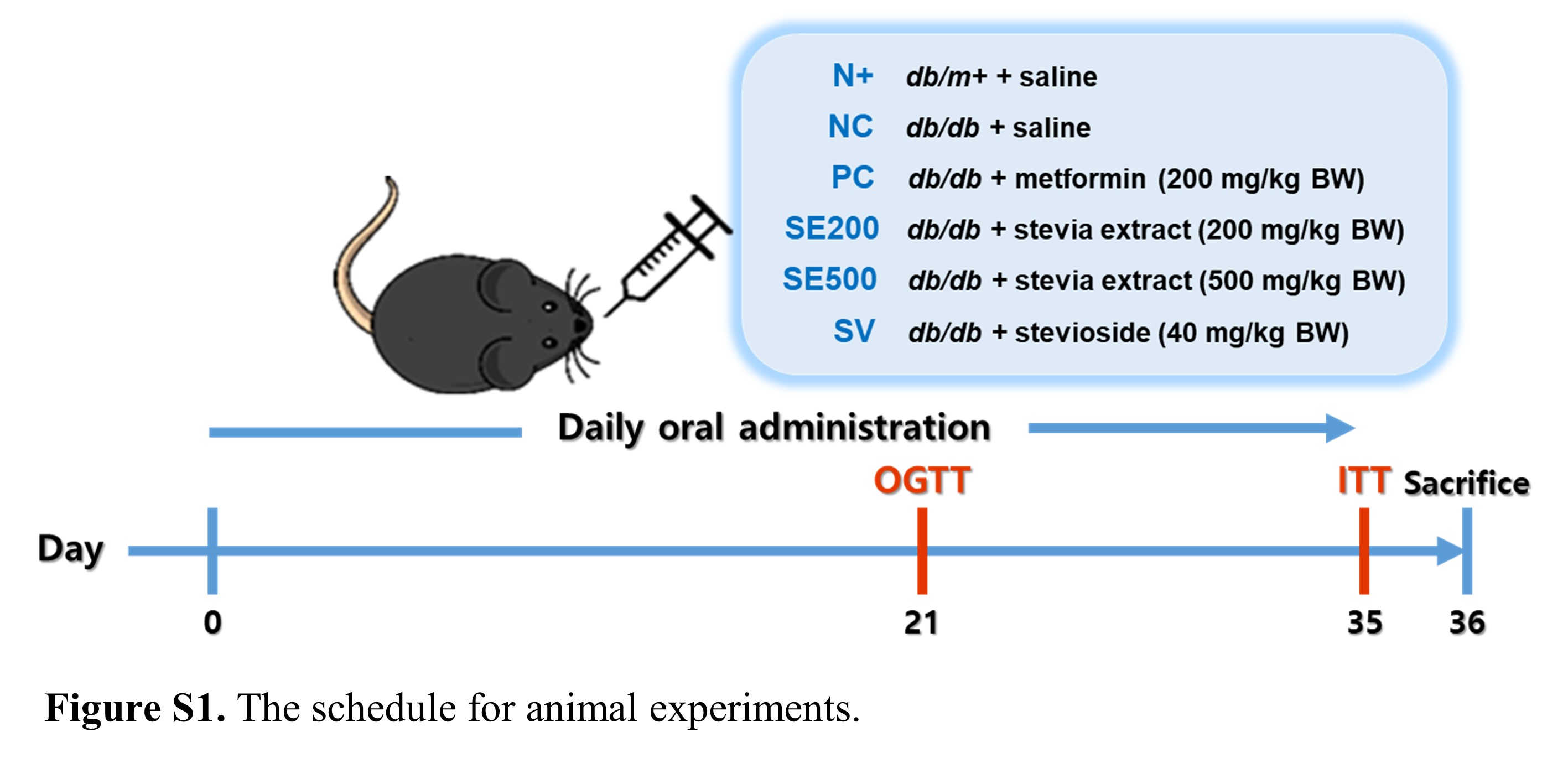

Supplement: Supplementary file 1 — Additional file 1. FigureS1. The schedule for animal experiments. [file 12906_2023_4033_MOESM1_ESM.jpg]

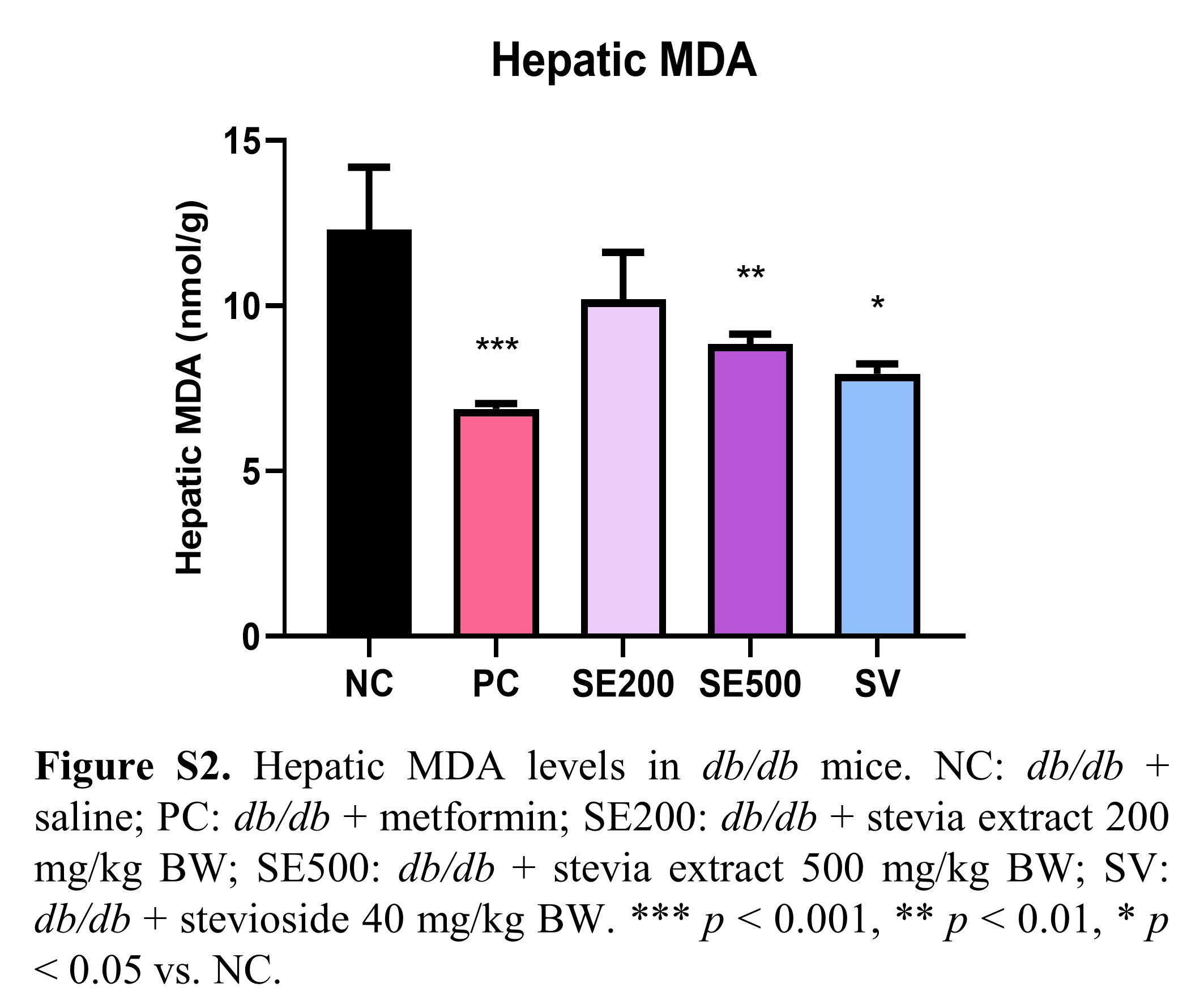

Supplement: Supplementary file 2 — Additional file 2. FigureS2. Hepatic MDA levels in db/db mice. [file 12906_2023_4033_MOESM2_ESM.jpg]
