# Supplementary material for: Stevia (Stevia rebaudiana) extract ameliorates insulin resistance by regulating mitochondrial function and oxidative stress in the skeletal muscle of db/db mice
Source: BMC Complement Med Ther. 2023 Jul 24;23:264. doi: 10.1186/s12906-023-04033-5 (PMC10367355; doi:10.1186/s12906-023-04033-5)

Original images for Blots - Figure 5.

N+ NC PC SE200 SE500 SV

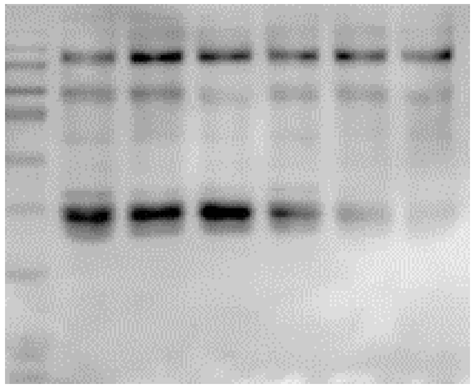

p-IRS
(180 kDa)

N+ NC PC SE200 SE500 SV

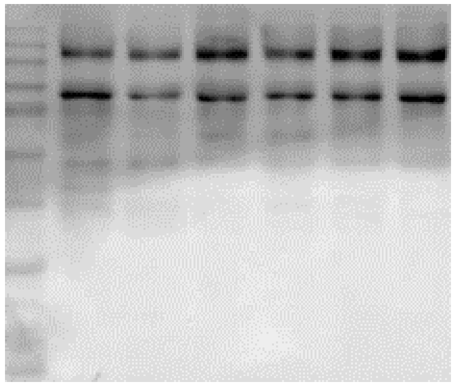

IRS
(180 kDa)

N+ NC PC SE200 SE500 SV

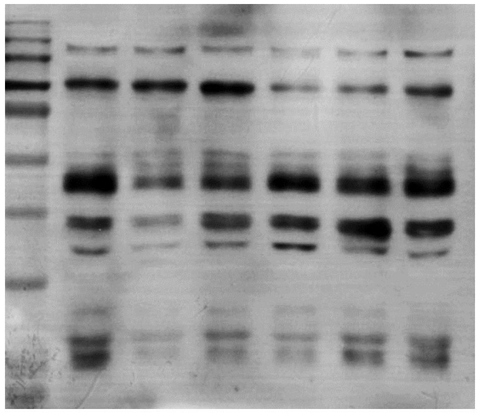

p-Akt
(60 kDa)

N+ NC PC SE200 SE500 SV

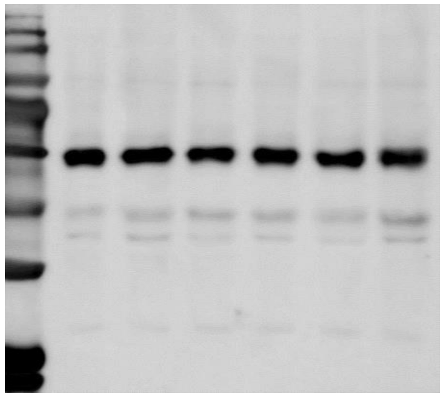

Akt
(60 kDa)

N+ NC PC SE200 SE500 SV

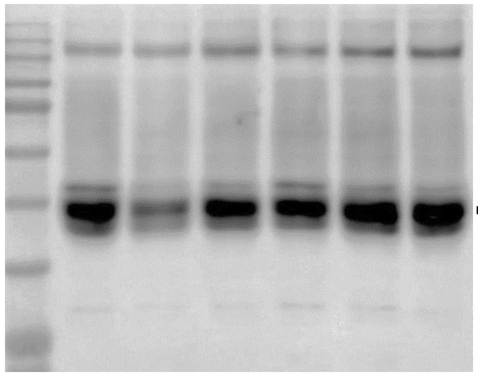

GLUT4
(50 kDa)

N+ NC PC SE200 SE500 SV

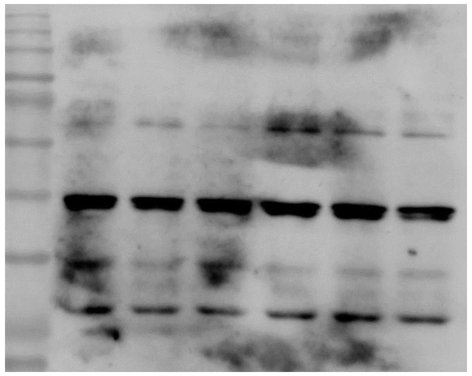

α-tubulin
(50 kDa)

Original images for Blots - Figure 6.

N+ NC PC SE200 SE500 SV

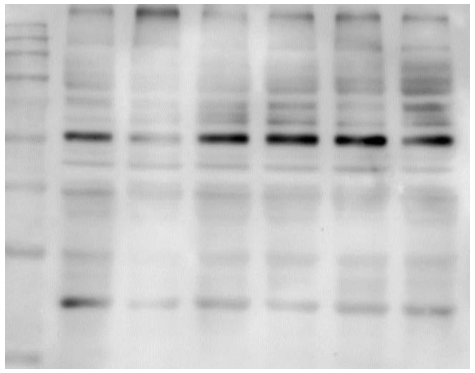

cytosolic
p-AMPKα
(62 kDa)

N+ NC PC SE200 SE500 SV

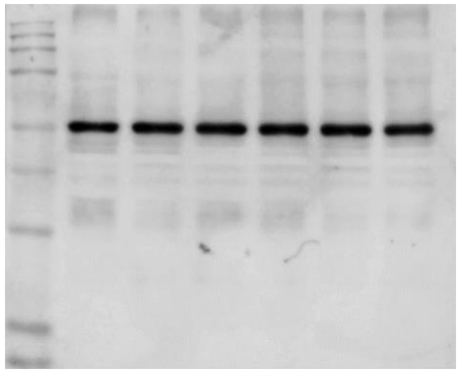

cytosolic
AMPKα
(63 kDa)

N+ NC PC SE200 SE500 SV

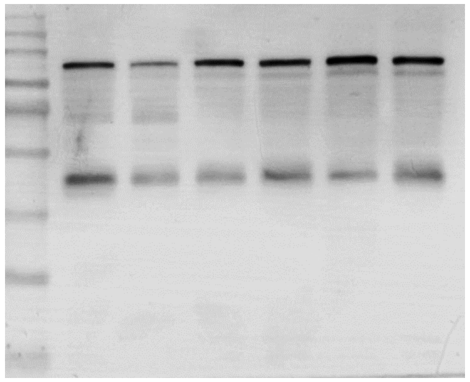

cytosolic
SIRT1
(121 kDa)

N+ NC PC SE200 SE500 SV

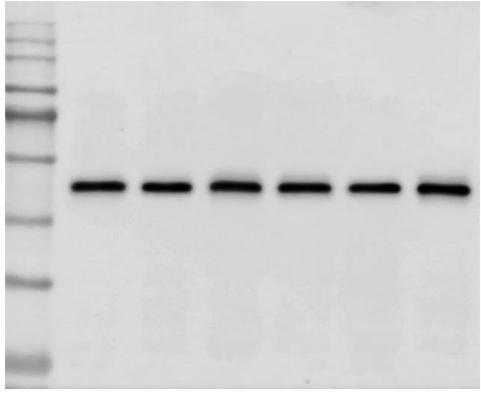

α-tubulin
(50 kDa)

N+ NC PC SE200 SE500 SV

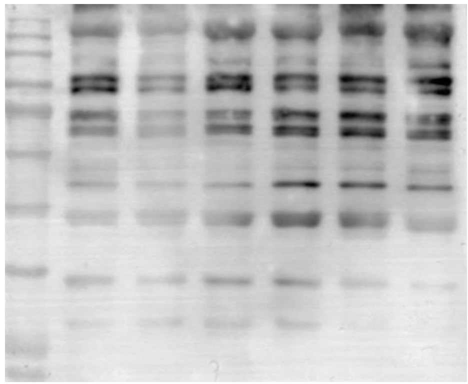

nuclear
SIRT1
(121 kDa)

N+ NC PC SE200 SE500 SV

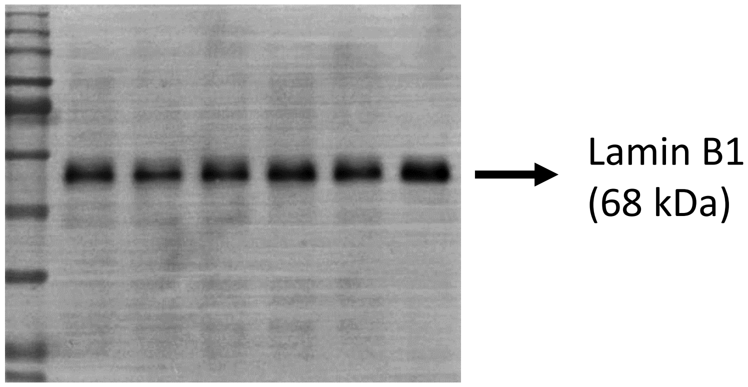

Lamin B1
(68 kDa)

Original images for Blots - Figure 6.

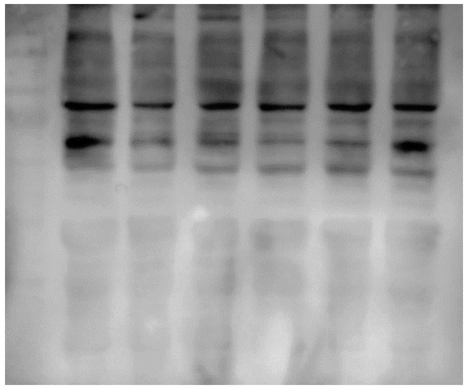

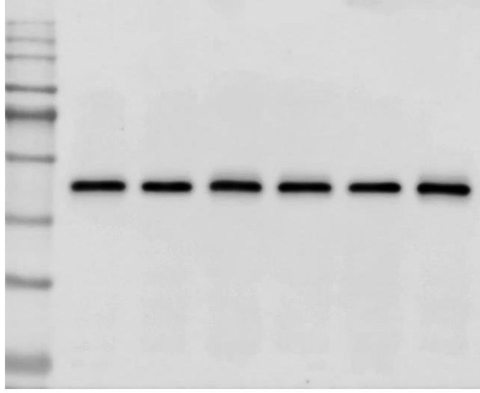

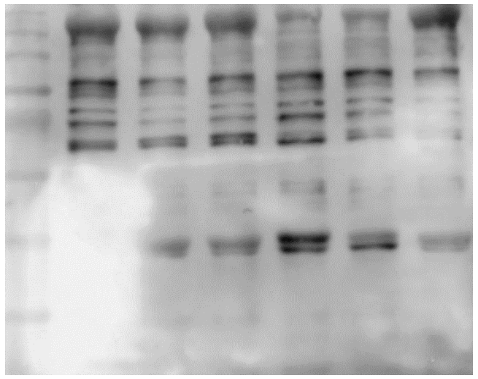

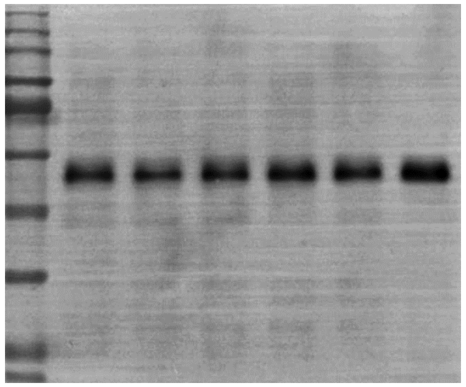

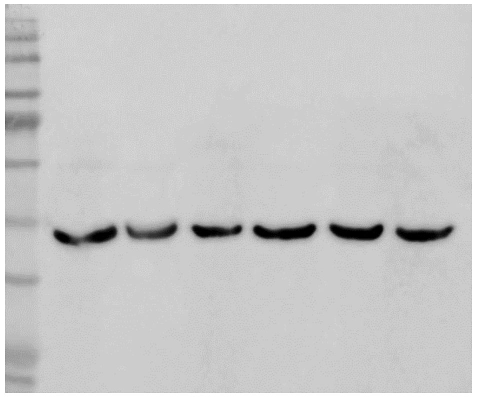

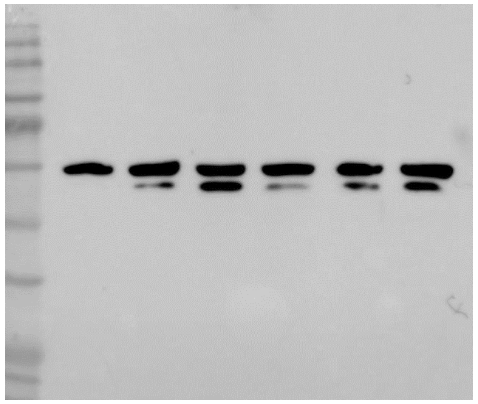

Original images for Blots - Figure 7.

N+ NC PC SE200 SE500 SV

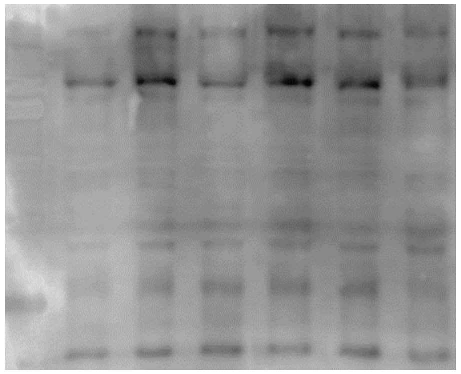

→ 4-HNE (100 kDa)

N+ NC PC SE200 SE500 SV

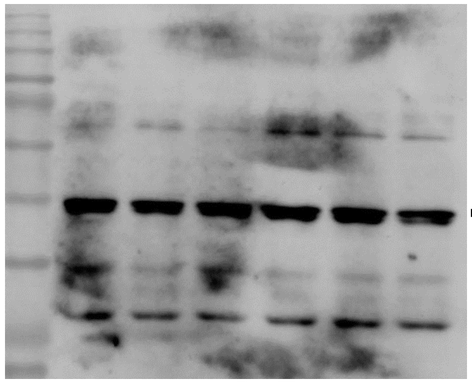

→ α-tubulin (50 kDa)

N+ NC PC SE200 SE500 SV

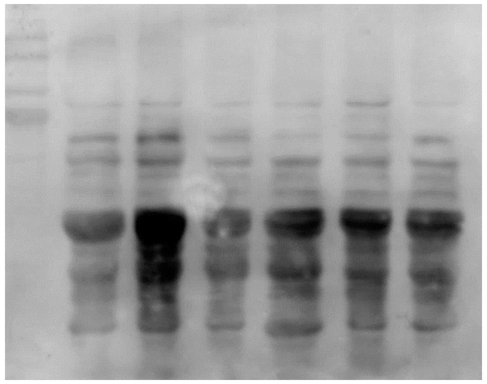

→ *cytosolic* HO-1 (32 kDa)

N+ NC PC SE200 SE500 SV

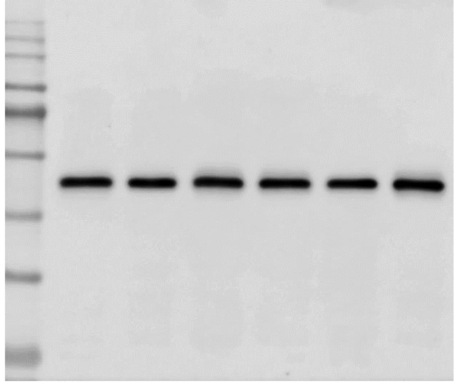

→ α-tubulin (50 kDa)

N+ NC PC SE200 SE500 SV

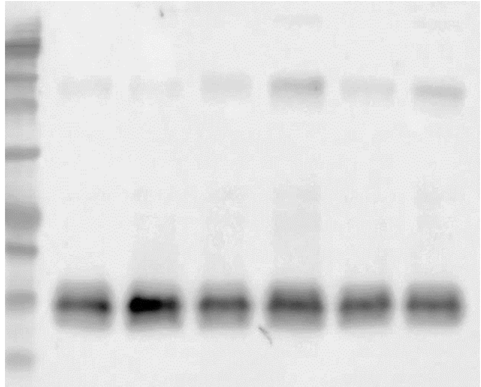

→ *cytosolic* SOD1 (20 kDa)

N+ NC PC SE200 SE500 SV

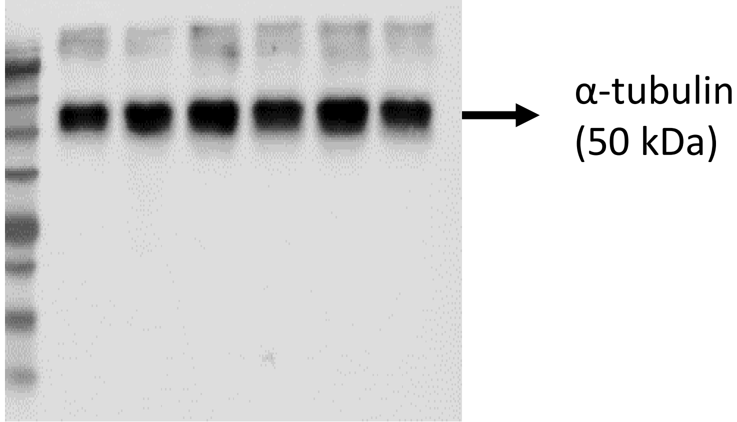

→ α-tubulin (50 kDa)

Original images for Blots - Figure 7.

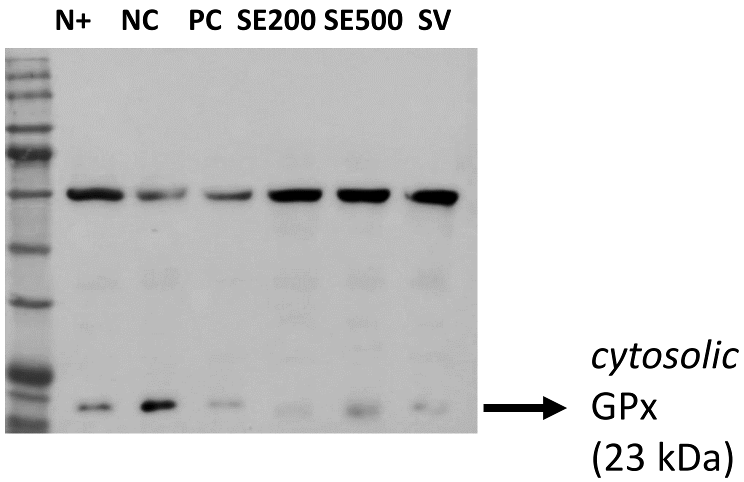

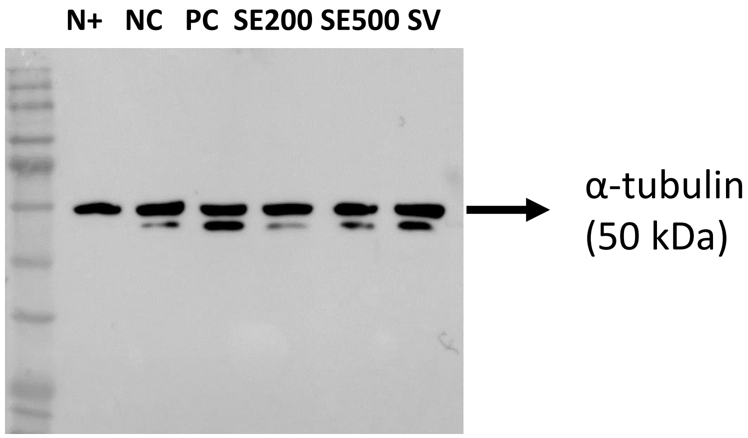

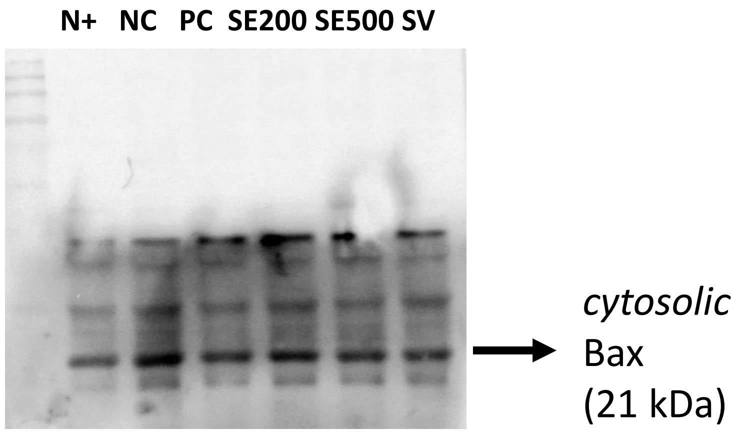

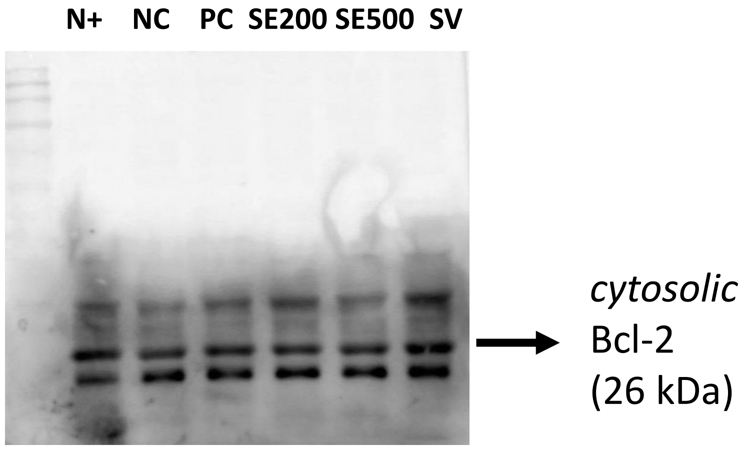

Supplement: Supplementary file 3 — Additional file 3. Original images for western blots. [file 12906_2023_4033_MOESM3_ESM.pdf]
